# IκBα polymorphism at promoter region (rs2233408) influences the susceptibility of gastric cancer in Chinese

**DOI:** 10.1186/1471-230X-10-15

**Published:** 2010-02-05

**Authors:** Shiyan Wang, Linwei Tian, Zhirong Zeng, Mingdong Zhang, Kaichun Wu, Minhu Chen, Daiming Fan, Pinjin Hu, Joseph JY Sung, Jun Yu

**Affiliations:** 1 Institute of Digestive Disease and Department of Meddicine and Therapeutics, Li Ka Shing Institute of Health Sciences, The Chinese University of Hong Kong, Hong Kong, China; 2 School of Public Health and Primary Care, The Chinese University of Hong Kong, Hong Kong, China; 3 Department of Gastroenterology, First Affiliated Hospital, Sun Yat-Sen University, Guangzhou, China; 4 State Key Laboratory of Cancer Biology and Institute of Digestive Diseases, Xijing Hospital, Fourth Military Medical University, Xi'an, China

## Abstract

**Background:**

Nuclear factor of kappa B inhibitor alpha (IκBα) protein is implicated in regulating a variety of cellular process from inflammation to tumorigenesis. The objective of this study was to investigate the susceptibility of rs2233408 T/C genotype in the promoter region of *IκBα *to gastric cancer and the association of this polymorphism with clinicopathologic variables in gastric cancer patients.

**Methods:**

A population-based case-control study was conducted between 1999 and 2006 in Guangdong Province, China. A total of 564 gastric cancer patients and 566 healthy controls were enrolled in this study. rs2233408 genotypes in *IκBα *were analyzed by TaqMan SNP genotyping assay.

**Results:**

Both rs2233408 T homozygote (TT) and T heterozygotes (TC and TT) had significantly reduced gastric cancer risk (TT: OR = 0.250, 95% CI = 0.069-0.909, *P *= 0.035; TC and TT: OR = 0.721, 95% CI = 0.530-0.981, *P *= 0.037), compared with rs2233408 C homozygote (CC). rs2233408 T heterozygotes were significantly associated with reduced risk of intestinal-type gastric cancer with ORs of 0.648 (95% CI = 0.459-0.916, *P *= 0.014), but not with the diffuse or mix type of gastric cancer. The association between rs2233408 T heterozygotes and gastric cancer appeared more apparent in the older patients (age>40) (OR = 0.674, 95% CI = 0.484-0.939, *P *= 0.02). rs2233408 T heterozygotes was associated with non-cardiac gastric cancer (OR = 0.594, 95% CI = 0.411-0.859, *P *= 0.006), but not with cardiac gastric cancer. However, rs2233408 polymorphism was not associated with the prognosis of gastric cancer patients.

**Conclusions:**

*IκBα *rs2233408 T heterozygotes were associated with reduced risk of gastric cancer, especially for the development of certain subtypes of gastric cancer in Chinese population.

## Background

Nuclear factor-κB (NF-κB) regulates many cellular functions including cell proliferation, apoptosis, angiogenesis, immune response, cell adhesion and differentiation [1]. NF-κB pathway plays important roles in linking inflammation to tumour development and progression in humans and mice [1]. Under unstimulated condition, NF-κB is inhibited by inhibitor nuclear factor-kappa B (IκB). Many oncogenes and carcinogens cause phosphorylation of IκB through the NF-κB inhibitor kinase (IKK) complex and proteasome-mediated degradation of IκB, leading to release of activated NF-κB [2]. The activated NF-κB system provides the cells with the production of growth factors as well as resistance to apoptotic and genotoxic insults, contributing to tumour growth and angiogenesis [1].

The IκB proteins include IκBα, IκBβ, IκBγ, IκBε, B-cell CLL/lymphoma 3 (BCL3), p105 and p100 [2]. *IκBα *gene, the main inhibitor of the NF-κB signaling, is located on chromosome 14q13 with 6 exons spanning approximately 3.5 kb. Polymorphisms in the promoter and exon region in the *IκBα *gene have been reported to be associated with inflammatory diseases and cancers [3-8]. rs696 AG genotype in the 3'-UTR of *IκBα *was associated with an increased risk of developing colorectal cancer in Chinese elderly population [3]. *IκBα *rs3138054 A/G (+1678) and rs2233419 C/T (+2025) polymorphism showed different frequency distributions between patients with multiple myeloma and controls [5]. Kim L *et al *reported that there was no significant association of *IκBα *polymorphisms with development of hepatocellular carcinoma among chronic hepatitis B patients [6]. *IκBα *-826 polymorphism and -826T/-550A/-519C haplotype were significantly associated increased risk of rheumatoid arthritis [7]. The frequency of haplotype GTC (-881G/-826T/-297C) was significantly higher among patients with acute respiratory distress syndrome in Caucasians [8]. However, the association of *IκBα *promoter polymorphism with gastric cancer is still unknown. We sequenced the 2 kb promoter region of *IκBα *on 32 gastric cancer tissues and 34 normal gastric tissues and found that rs2233408 polymorphism showed bigger difference of the distribution between gastric cancer patients as compared with the control subjects. Thus, we conducted a large-scale case-control study on Chinese population to investigate the association of rs2233408 polymorphism with gastric cancer or clinicopathologic variables of gastric cancer patients.

## Methods

### Subjects

A population-based case-control study was conducted between 1999 and 2006 in Guangdong Province, China. The case series included 1010 Chinese gastric cancer patients, diagnosed in the First Affiliated Hospital of Sun Yat-sen University, Guangzhou and the Oncology Hospital, Guangzhou from January 1999 to December 2006. Diagnosis was confirmed histologically as gastric cancer. The median duration of follow-up was 14 months (ranging from 0 to 98 months). Clinical data of patients were collected from medical record and structured interview of patients using a questionnaire. Because 187 of the patients were transferred to other hospitals with their medical history records or unwilling to complete the questionnaires, some clinical data of patients was unable to be traced, leaving a total of 823 with sufficient clinical information available. Since the DNA samples of gastric cancer were extracted from paraffin-embedded tissues, 564 gastric cancer cases with both good DNA quality and sufficient clinical data were selected for genotyping. All patients diagnosed to have gastric cancer received surgical resection of the tumor. The control series included 1500 Cantonese healthy subjects from the general population of Guangzhou in southern China. Among them, 566 control subjects were randomly selected, frequency matched to the case group by age and gender. There are 373 males (66%) and 191 females (34%) in 564 gastric cancer cases whereas there are 354 males (63%) and 212 females (37%) in 566 control subjects. In the literature, the percentage of rs2233408 TC/TT genotype in Asian populations ranged from 11% to 21% [6,7]. With the sample size in the present study, we have a study power of 76-93% to detect an OR of 1.5 or 0.7 at a significance level of 0.05. Patients and control subjects gave informed consent for participation in this study and the study protocol was approved by the Clinical Research Ethics Committee of the Sun Yat-sen University of Medical Sciences.

### DNA extraction

Genomic DNA was extracted from paraffin-embedded tissues of 1010 gastric cancer patients using QIAamp DNA Mini Kit (Qiagen, Germany) and from 2 ml peripheral blood of 1500 healthy controls using Gentra Puregene Blood Kit (Gentra Systems, Inc., Minneapolis, MN) All extracted DNA was resuspended in UltraPure RNAse/DNAse-Free Distilled water (Invitrogen, Carlsbad, CA).

### IκBα genotyping

IκBα genotyping was performed by TaqMan(R) allelic discrimination using predesigned Custom TaqMan SNP Genotyping Assays kit (Applied Biosystems). The primers and probes for rs2233408 genotyping are 5'-GAAACACCGGCGCGG-3', 5'-TTTGCTTTCCCCAGACTTCTAAGG-3', 5'-CTGCAGCCTCCTAAC-3' (VIC), 5'-TGCAGCCCCCTAAC-3' (FAM). PCRs were run in a 20 μl reaction solution containing 200 ng of template DNA, 10 μl TaqMan Universal PCR Master Mix No AmpErase UNG (2×) (Applied Biosystems) and 0.5 μl 40×SNP Genotyping Assay (Applied Biosystems). PCR was performed at 95°C for 10 min and 40 cycles at 92°C for 15 sec and 60°C for 1 min. The samples were amplified, read and analyzed in Applied Biosystems 7500 Real-Time PCR System (Applied Biosystems).

### Statistics

Hardy-Weinberg equilibrium of alleles at *IκBα *loci was assessed by χ^2 ^tests. The effects of the genotypes at *IκBα *loci on the risk of gastric cancer were represented as odds ratios (OR) with 95% confidence interval (CI) by logistic regression. OR estimates for Lauren types and tumor location were adjusted for age and gender. OR estimates in age groups were adjusted for gender. Relationships between *IκBα *polymorphism and clinicopathologic characteristics of gastric cancer patients were compared using contingency tables and Pearson's χ^2 ^test. Kaplan-Meier survival curves and the log-rank test for trend were used to evaluate the relationship between *IκBα *polymorphisms and the prognosis from the date of primary diagnosis to the end of follow-up. The primary outcome is the overall survival of gastric cancer patients. The multivariate Cox regression analysis was performed to assess the prognostic value of *IκBα *polymorphisms with adjustment for age, gender, tumor size grade, lymph node status and distant metastasis. Hazard ratios (HR) and 95% CI for each factor in multivariate analysis were calculated from the Cox regression model. Differences were regarded significant at *P *< 0.05. All the above statistical analyses were performed using SAS software (SAS Institute, Inc., Cary, North Carolina).

## Results

### IκBα polymorphism was associated with reduced risk of gastric cancer

Before genotyping, we conducted Hardy-Weinberg equilibrium analysis of rs2233408 polymorphism. We found that the alleles of rs2233408 polymorphism were in Hardy-Weinberg equilibrium with non-significant χ^2 ^values (*P *= 0.25). Analysis of rs2233408 genotype polymorphism showed a significant difference between gastric cancer patients and controls (Table 1). Gastric cancer patients had significantly lower carriage rate of rs2233408 TT genotype and T heterozygotes (TC and TT) with reduced ORs of 0.250 (95% CI = 0.069-0.909, *P *= 0.035) and of 0.721 (95% CI = 0.530-0.981, *P *= 0.037) than controls (Table 1), suggesting a dominative effect of T allele. The allele frequency of rs2233408 T was significantly reduced in gastric cancer patients compared with that of the controls (OR = 0.696, 95% CI = 0.523-0.925, *P *= 0.013) (Table 1).

**Table 1:** Adjusted Odds Ratios (ORs) and 95% Confidence Intervals (CIs) for gastric cancer in relation to *IκBα *rs2233408 genotypes and alleles

	Controls (%)	Cases (%)	OR* (95%CI)	*P*-value
**Genotype**				
**CC**	451 (79.7)	476 (84.4)	1	
**TC**	104 (18.4)	85 (15.1)	0.771 (0.562-1.059)	0.108
**TT**	11 (1.9)	3 (0.5)	0.250 (0.069-0.909)	0.035
**TC/TT**	115 (20.3)	88 (15.6)	0.721 (0.530-0.981)	0.037
**Allele**				
**C**	1006 (88.9)	1037 (91.9)	1	
**T**	126 (11.1)	91 (8.1)	0.696 (0.523-0.925)	0.013

[NOTE] *Adjusted for age and gender.

### IκBα polymorphism was associated with intestinal-type gastric cancer

We evaluated the role of rs2233408 polymorphism in different subtypes of gastric cancer. Stratified by the Lauren classification, rs2233408 overall T carriers (TC and TT) were also significantly lower in patients of intestinal type gastric cancer (OR = 0.648, 95% CI = 0.459-0.916, *P *= 0.014), as compared with rs2233408 CC genotype (Table 2). However, there was no significant association of rs2233408 genotypes with mix type or diffuse type gastric cancer probably due to the small number of diffuse/mix type gastric cancer cases (Table 2).

**Table 2:** Adjusted Odds Ratios (ORs) and 95% Confidence Intervals (CIs) for gastric cancer, stratified by Lauren type, in relation to *IκBα *rs2233408 genotypes

Genotype		Intestinal			Diffuse			Mix		
		
	Control	Cases	OR* (95% CI)	*P*-value	Cases	OR* (95% CI)	*P*-value	Cases	OR* (95%CI)	*P*-value
**CC**	451	354	1		27	1		64	1	
**TC/TT**	115	59	0.648 (0.459-0.916)	0.014	2	0.289 (0.068-1.237)	0.094	23	1.404 (0.835-2.359)	0.201

[NOTE] *Adjusted for age and gender. Among 564 patients, 35 patients have missing data on Lauren classification.

### Association between IκBα rs2233408 polymorphism and age-at-onset of gastric cancer

The roles of rs2233408 polymorphism in gastric cancer in different age groups were evaluated. Since gastric cancer in younger patients under the age of 40 showed a different genetic background and pathogenic mechanism of disease from conventional gastric cancers [9-12], the subjects were divided into two age groups (age < = 40 and age >40). In the age group of >40, rs2233408 overall T carriers (TC and TT) remained to be significantly associated with gastric cancer compared with rs2233408 CC genotype (OR = 0.674, 95% CI = 0.484-0.939, *P *= 0.020) (Table 3). However, the associations of this polymorphism was not statistically significant in the younger age group (age <40) (Table 3).

**Table 3:** Adjusted Odds Ratios (ORs) and 95% Confidence Intervals (CIs) for gastric cancer in relation to *IκBα *rs2233408 genotypes, stratified by age groups

	Age ≦ 40				Age >40			
	
Genotype	Controls	Cases	OR* (95% CI)	*P*-value	Controls	Cases	OR* (95% CI)	*P*-value
**CC**	91	52	1		360	424	1	
**TC/TT**	18	11	1.033 (0.449-2.377)	0.939	97	77	0.674 (0.484-0.939)	0.020

[NOTE] *Adjusted for gender.

### IκBα polymorphism was associated with non-cardiac gastric cancer

We evaluated whether the association between rs2233408 polymorphism and gastric cancer was modified by the tumor location: cardiac or non-cardiac gastric cancer. rs2233408 overall T carriers (TC and TT) showed lower prevalence in non-cardiac gastric cancer than in controls (OR = 0.594, 95% CI = 0.411-0.859, *P *= 0.006) (Table 4). However, no significant difference was observed between cardiac gastric cancer patients and controls.

**Table 4:** Adjusted Odds Ratios (ORs) and 95% Confidence Intervals (CIs) for gastric cancer, stratified by tumor location, in relation to *IκBα *rs2233408 genotypes

Genotype		Cardiac			Non-cardiac		
		
	Controls	Cases	OR* (95% CI)	*P*-value	Cases	OR* (95% CI)	*P*-value
**CC**	451	121	1		310	1	
**TC/TT**	115	32	1.021 (0.649-1.607)	0.927	47	0.594 (0.411-0.859)	0.006

[NOTE] *Adjusted for age and gender. Among 564 patients, 54 patients have missing data on tumor location.

### Association of IκBα polymorphism with H. pylori infection

To evaluate whether *H. pylori *prevalence was influenced by IκBα rs2233408 polymorphisms, the distributions of *H. pylori *infection between rs2233408 genotypes were examined (Table 5). There was no significant association of rs2233408 genotypes with *H. pylori *infection.

**Table 5:** The distribution of *IκBα *rs2233408 polymorphisms in gastric cancer patients

Genotype	*H. pylori *infection		*P*-value
		
	Negative (%)	Positive (%)	
**CC**	143 (88%)	90 (86%)	0.576
**TC/TT**	19 (12%)	15 (14%)	

### IκBα polymorphism was not associated with the survival of gastric cancer patients

**Figure 1:**
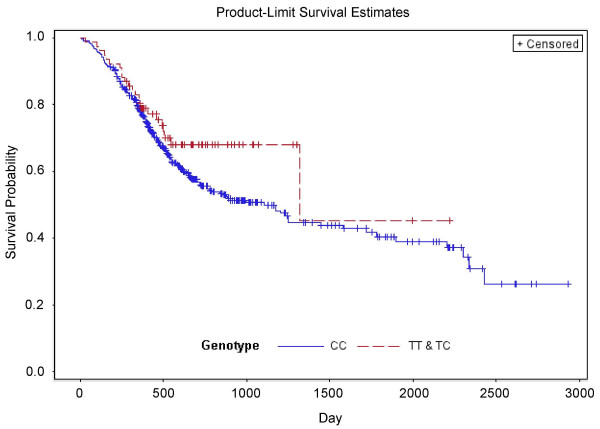
**Kaplan-Meier survival curves show that overall survival of gastric cancer patients is not associated with *IκBα *rs2233408 genotypes**. *P *value was calculated using a log-rank test.

Kaplan-Meier survival curves showed that overall survival of the gastric cancer patients were not significantly different among different rs2233408 genotypes (Figure 1). In the multivariate Cox regression analysis, tumor size grade, lymph node status, and distant metastasis were significantly associated with the survival of gastric cancer patients (Table 6). However, rs2233408 genotypes were not associated with the outcome in gastric cancer patients (Table 6).

**Table 6:** Cox multivariate regression analysis of potential factors for overall survival in gastric cancer patients

Variable	HR (95% CI)	*P*-value
Age	1.01 (0.99-1.02)	0.44
Gender		
Male	1	
Female	0.99 (0.71-1.38)	0.96
Tumor staging		
T1	1	
T2	4.49 (0.56-36.25)	0.16
T3	7.44 (1.00-55.67)	0.05
T4	10.01 (1.31-76.70)	0.03
Lymph node		
Negative	1	
Positive	2.52 (1.52-4.20)	0.0004
Metastasis		
No	1	
Yes	2.94 (1.96-4.40)	< .0001
Lauren Classification		
Intestinal	1	
Diffuse or Mix	0.92 (0.54-1.58)	0.76
Location		
Cardiac	1	
Non-Cardiac	0.91 (0.61-1.34)	0.62
Differentiation		
Poor	1	
Moderate or Well	1.24 (0.81-1.90)	0.31
rs2233408 genotype		
CC	1	
TC/TT	0.69 (0.42-1.12)	0.13

## Discussion and Conclusions

In the present study, *IκBα *rs2233408 T heterozygotes and homozygote were significantly associated with reduced risk of gastric cancer in southern Chinese population. This result was consistent with the report by Lin *et al *who demonstrated that rs2233408 T allele was more prevalent in healthy controls than in patients with rheumatoid arthritis probably due to different transcriptional activities of IκBα and the consequent activity of NF-κB [7].

We further demonstrated that *IκBα *rs2233408 polymorphism was associated with reduced risk of intestinal type gastric cancer rather than the diffuse-type. It has been known that the development of the intestinal-type malignancy is a longer multistep process via gastric atrophy, intestinal metaplasia, dysplasia and ultimately intestinal-type carcinoma. However, diffuse-type gastric cancer occurred without the intermediate steps. NF-κB expression in gastric tissues increased with the progression of precancerous gastric lesions including intestinal metaplasia, dysplasia to gastric carcinoma [13]. Thus, IκBα/NF-κB pathway seemed more involved in the progression of the intestinal-type carcinoma. In addition, bile acid induced NF-κB-dependent the intestine-specific transcription factor caudal type homeobox 2 (Cdx-2) expression and Cdx-2 downstream target guanylyl cyclase C, which promoted the malignant transition from inflammation to metaplasia carcinoma [14]. Cdx-2 is intestine-specific transcription factor mediating the extracellular matrix-mediated intestinal cell differentiation and is associated with intestinal-type gastric cancer [15-19]. Thus one possible explanation for the association of *IκBα *polymorphism with intestinal-type gastric cancer was that different production of NF-κB inhibitor IκBα mediated by *IκBα *polymorphism led to different NF-κB activation and downstream Cdx-2 expression, promoting transition from inflammation, intestinal metaplasia to carcinoma.

On the other hand, since the number of diffuse/mix type gastric cancer is small, we could not make an affirmative conclusion on association between rs2233408 polymorphism and diffuse/mix gastric cancer. Therefore future studies are warranted to examine the association of rs2233408 polymorphism with diffuse/mix gastric cancer by collecting new samples from patients with diffuse/mix type gastric cancer.

*IκBα *rs2233408 polymorphism was also associated with elderly gastric cancer patients, but was not associated with early-onset gastric cancers. Early onset and conventional gastric cancer appears to have a different genetic background and different pathogenic disease mechanism [12,20]. Milne AN *et al *found that early-onset gastric cancers displayed a COX-2 low, trefoil factor-1-expressing phenotype, whereas COX-2 over-expression and loss of trefoil factor-1 was found in conventional cancers [20]. We also found IL-1B-511 polymorphism was associated with gastric cancer in elderly group rather than younger group of patients [21]. These findings suggested that the transition from pre-cancerous lesions to malignancy of multi-steps gastric carcinogenesis was a lengthy process, in which pro-inflammatory factors such as COX-2, IL-1B and IκBα may be involved.

*IκBα *rs2233408 polymorphism was found to be associated with non-cardiac cancer but not cardiac cancer. Non-cardiac cancer rather than cardiac cancer were consistent with the current view that the development of malignancy is a long multistep process from chronic gastritis induced by *H. pylori *infection, atrophic gastritis with loss of acid secretion, dysplasia and finally to cancer [22]. Atrophy, *H. pylori *infection and hypochlorhydira were associated with increased risk of non-cardiac gastric cancer but protected against cardia and esophageal adenocarcinomas [23]. Proinflammatory cytokine gene polymorphisms including interleukin-1β, interleukin-1 receptor antagonist, interleukin-10 and tumor necrosis factor-A increased the risk of non-cardiac gastric adenocarcinoma but not cardia and esophageal adenocarcinomas [23]. These findings collectively suggested that non-cardiac cancer is genetically different with the cardiac cancer.

The association of *IκBα *rs2233408 polymorphism with gastric cancer patient outcome was also evaluated. Patients with *IκBα *rs2233408 T heterozygotes did not show better prognosis than those with C homozygote as determined by Kaplan-Meier analysis or by multivariate Cox regression analysis, suggesting that *IκBα *rs2233408 polymorphism did not affect gastric cancer out come. Other factors, including tumor size grades, lymph node status and distant metastasis, were significantly correlated with the survival of gastric cancer patients.

In conclusion, rs2233408 T heterozygotes had significantly reduced gastric cancer risk, compared with rs2233408 C homozygote genotype in southern Chinese. In addition, there is heterogeneity in the risk of different sub-types for gastric cancer susceptibility in *IκBα *polymorphism. rs2233408 polymorphism was associated with increased risk of intestinal-type, non cardiac and elderly gastric cancers.

## Abbreviations

BCL3: B-cell CLL/lymphoma 3; Cdx-2: caudal type homeobox 2; COX-2: cyclooxygenase-2; *H. Pylori*: *Helicobacter pylori*; IκB: inhibitor nuclear factor-kappa B; IKK: Nuclear factor NFkappaB inhibitor kinase; IL-1B: interleukin 1 beta; NF-κB: nuclear factor kappa B.

## Competing interests

The authors declare that they have no competing interests.

## Authors' contributions

SW performed experiments and drafted the paper. LT did statistical analysis. ZZ, MC and PH collected samples and data; MZ designed the research; KW, DF, JJYS commented on the study and revised the paper; JY designed research, analyzed data and revised the paper. All the authors read and approved the final manuscript.

## Pre-publication history

The pre-publication history for this paper can be accessed here:

http://www.biomedcentral.com/1471-230X/10/15/prepub
